# Quality of life and associated factors among primary caregivers of children and adolescents with neurodevelopmental disorders attending public hospitals in Addis Ababa, Ethiopia: a cross-sectional study

**DOI:** 10.1093/inthealth/ihae055

**Published:** 2024-09-24

**Authors:** Jerman Dereje, Abenet Kassaye, Abiy Mulugeta, Girmaw Medfu, Shegaye Shumet, Tilahun Kassew

**Affiliations:** Department of Psychiatry, College of Health and Medical Science, Haramaya University, P.O. Box 235, Harar, Ethiopia; Department of Psychiatry Nursing, College of Medicine and Health Science, Wollo University, P.O. Box 1145, Dessie, Ethiopia; Department of Psychiatry, College of Health and Medical Science, Haramaya University, P.O. Box 235, Harar, Ethiopia; Department of Psychiatry, College of Medical and Health Science, University of Gondar, P.O. Box 196, Gondar, Ethiopia; Department of Psychiatry, College of Medical and Health Science, University of Gondar, P.O. Box 196, Gondar, Ethiopia; Department of Psychiatry, College of Medical and Health Science, University of Gondar, P.O. Box 196, Gondar, Ethiopia

**Keywords:** Addis Ababa, Ethiopia, neurodevelopmental disorders, primary caregivers, quality of life

## Abstract

**Background:**

Neurodevelopmental disorders are a set of disorders that negatively affect the acquisition of skills in a variety of developmental domains, including motor function, learning, socialization, language and cognition. However, there is no information available on the standard of living of Ethiopian primary caregivers of children and adolescents with neurodevelopmental disorders. Therefore, this study aimed to assess the quality of life (QOL) and associated factors among primary caregivers of children and adolescents with neurodevelopmental disorders in Addis Ababa, Ethiopia.

**Methods:**

A cross-sectional study was conducted during 1–30 May 2022. Systematic random sampling was used to obtain 352 samples. QOL was measured using the WHO Quality of Life Brief. The gathered information was coded, entered into EpiData 4.6.0.2 and analyzed with SPSS version 26. Multiple linear regression analysis was used to identify the correlates of QOL and the strength of the correlation was measured by β coefficient with 95% CI.

**Results:**

The mean score of the overall QOL was 62.61 with a SD of 5.17. The mean (±SD) scores for the physical health, psychological, environmental and social relationship domains of primary caregivers were 57.36±9.98, 66.98±9.39, 66.06±12.91 and 60.02±9.14, respectively. Age was significantly associated with physical domain (β=−0.25, 95% CI −0.43 to −0.07) and with environmental domain (β=−4.57, 95% CI −9.06 to −0.09). Being divorced/widowed was negatively associated with psychological health (β=−2.99, 95% CI −5.82 to −0.17) and social health (β=−0.62, 95% CI −1.33 to −0.10). The presence of medical illness was negatively associated with the physical health domain (β=−4.32, 95% CI −7.64 to −2.91) and the environmental domain (β=−3.11, 95% CI −5.71 to −0.51). Poor social support was negatively associated with psychological health (β=−3.25, 95% CI −5.89 to −0.61) and the social health domain (β=−3.39, 95% CI −11.3 to 4.6), and moderate social support (β=8.62, 95% CI 3.15 to 14.09) was positively associated with physical health. Depression (β=−6.32, 95% CI −11.96 to −0.67) and anxiety (β =−3.07, 95% CI −5.80 to −0.34) were negatively associated with physical health and the psychological health domain, respectively.

**Conclusions:**

The findings from this study indicate that all dimensions of QOL of primary caregivers of children and adolescents with neurodevelopmental disorders in this study setting were compromised. Being divorced or widowed, lack of formal education, age, average monthly income, poor social support, depression, anxiety and the presence of medical illness were factors associated with QOL in all domains. This requires integrating a bio-psychosocial perspective, a positive mental health strategy and pharmaceutical therapies to enhance QOL for caregivers of children and adolescents with neurodevelopmental disorders.

## Introduction

The term ‘neurodevelopmental disorders’ (NDDs) refers to a group of conditions in which the growth of the central nervous system is hampered.^1^ This may affect how children and adolescent develop their abilities in several developmental domains, such as motor control, learning, sociability, language and cognition.^2^ The most frequently diagnosed NDDs include autism spectrum disorders, attention deficit hyperactivity disorders, global developmental delay, intellectual disability and learning disabilities.^3^

Quality of life (QOL) is defined as an individual's view of their place in life about their objectives, expectations, standards and worries, as well as the culture and value systems in which they live. It encompasses a wide range of factors, including a person's physical and mental well-being, level of independence, social connections, religious views and relationship to important environmental elements.^4^

QOL for children and adolescents with NDDs is significantly impacted, as is QOL for the primary caregivers of these individuals.^5^ Because of the chronic nature of the diseases, which force family members to provide primary care at home, the problem no longer just impacts children and adolescents who have been diagnosed, it now affects the entire family.^6^

Approximately 15% of the world’s population, or 95 million people, have NDDs, according to the WHO. These individuals are children and adolescents aged 0–17 y.^7^ The overall trend of children and adolescents is upward, which makes it more difficult for primary caregivers to provide childcare. According to earlier research, primary caregivers of children and adolescents with NDDs experienced greater levels of stress in comparison with the general population,^8^ as well as depression, anxiety and low satisfaction with life,^9^ resulting in a detrimental effect on their health-related QOL.^10^

Parenting children and adolescents with NDDs can be difficult, and it impacts both the general QOL and its various aspects. Studies conducted all around the world demonstrated that the four domains of primary caregivers of children and adolescents with NDDs were impacted, as well as the overall QOL of those caregivers. In the USA, the mean score for overall QOL was 56.19; in Brazil, it was 61.3; in Dhaka, Bangladesh, it was 43.28; in Pakistan, it was 65.30; in South Africa, it was 50.50; and in Nairobi, Kenya, it was 31.0.^11–16^

On the other hand, a variety of factors were identified by primary caregivers of children and adolescents with NDDs as being significant factors affecting their QOL. The QOL of primary caregivers was influenced by advanced age, divorce or widowhood, monthly income, educational level, employment position, financial and social support, as well as medical conditions such as depression and anxiety.^17–21^ Primary caregivers of people with various mental disorders have received substantially more attention than those who care for those with NDDs; despite this, studies assessing QOL and associated variables among primary caregivers of children and adolescents with NDDs have not been conducted in Ethiopia. Therefore, the goal of the current study was to assess the QOL and associated factors among primary caregivers of children and adolescents attending the outpatient departments at Yekatit 12 Hospital Medical College (Y12HMC) and St. Paul's Hospital Millennium Medical College (SPHMMC) in Addis Ababa, Ethiopia.

## Materials and methods

### Study settings, design and period

A cross-sectional study was carried out at Y12HMC and SPHMMC, both of which are in Ethiopia's capital city of Addis Ababa. These hospitals serve >5 million individuals in their catchment areas. These hospitals are currently teaching hospitals. Outpatient pediatric and adolescent psychiatric services are provided. During the country's working days, children and adolescent psychiatric institutions provide care for children and adolescents who have NDDs. Daily, about 15–20 children and adolescents receive follow-up treatment in the outpatient departments of the children and adolescent psychiatric clinics in both hospitals. Monthly, about 700 children and adolescents attend follow-up appointments at the psychiatry clinics in the outpatient departments in both hospitals. The data were collected from 1 to 30 May 2022. Given that cross-sectional studies are observational studies, the methodological design was constructed following Strengthening the Reporting of Observational Studies in Epidemiology (STROBE) standards, and the manuscript is organized in compliance with these guidelines.

### Source population, study population and eligibility criteria

The source populations were all primary caregivers who were ≥18 years old and had follow up for their children and adolescents at Yekatit 12 Hospital Medical College and St. Paul's Hospital Millennium Medical College. Children and adolescents under the age of eighteen who had a clinical diagnosis of neurodevelopmental disorder (NDD) confirmed by a family medicine specialist, child psychiatrist, or pediatrician at least six months before the study’s start were included in the study. The concept is primary caregivers should be greater than or equal to 18 years and their children and adolescents should be less than 18 years to be included in the study.

### Study variables

#### Outcome variable

The dependent variables were QOL and each of the four domains was calculated using the 26-item WHO Quality of Life Brief (WHOQOL-BREF) questionnaire as a continuous outcome. It has four domains (physical health, psychological, environmental and social) that denote an individual's perception of QOL and it also contains one perceived QOL item and one general health satisfaction status item.

#### Independent variables

The independent variables were the primary caregiver's sociodemographics (age, sex, monthly income, employment status, marital status and educational status), social factors (social support), primary caregiver clinical-related factors (depression, anxiety and chronic medical conditions), children and adolescents (age, sex, types of diagnosis, duration of disease, age at intervention).

### Sample size determination and sampling procedure

The required minimum sample size was determined using the single mean population formula. The mean and SD were taken from a study performed in Egypt (physical health domain QOL mean 43.20 with SD 9.13).^22^ The assumptions were margin of error (d=1); physical domain SD 9.13; Z-value at a=0.05 of 1.96, and adding a 10% non-response rate. Therefore,


\begin{eqnarray*}
{\mathrm{Minimum\,\, sample\,\, size }}\left( {\mathrm{n}} \right) = \frac{{{{{({\mathrm{Z}}\frac{\alpha }{2})}}^2}}}{{{{{\left( d \right)}}^2}}}{{\left( S \right)}^2}
\end{eqnarray*}

n=(1.96)²×(9.13)²=320

(1)^2^

Taking a non-response rate of 10%, then 320×10%=32.

Therefore, the minimum sample size required for this study was 352, adding the 10% non-response rate. Study participants were recruited using a systematic sampling technique to include primary caregivers who met the inclusion criteria as part of the sample.

### Data collection tool and procedure

Data were collected by face-to-face interviews using a structured and pretested questionnaire. The data collection tools were initially created in English, then translated into Amharic, a local language, then back into English to ensure the questionnaire's clarity. Data were collected by three Bachelor of Nursing professionals supervised by one psychiatry professional with the facilitation of the principal investigator. The supervisor distributed all the necessary items for the data collection on each data collection day and was tasked with checking the filled-in questionnaires for completeness and solving forwarded problems in a timely fashion during data collection. All the primary caregivers were approached by researchers during the data collection period, and those who met the criteria for eligibility were invited to take part in the study. Four participants were excluded from a total of 352, thus 348 participants were recruited for the study. Two participants were excluded from the study because they were unable to provide signed informed consent and two others were unable to communicate during the data collection period due to serious illnesses.

The questionnaire comprised seven sections. The first section consisted of sociodemographic variables, including age, sex, religion, ethnicity, marital status, educational status, occupational status and income. These sociodemographic characteristics were assessed using questions developed from the literature.

The second section concerned QOL, which was assessed using the WHOQOL-BREF. The WHOQOL-BREF comprises 26 items and is a self-administered generic questionnaire. It is a short version of the WHO QoL-100 scale. This tool is a sound, cross-culturally valid assessment of QOL and has proven to be a suitable tool for the assessment of QOL of primary caregivers of children and adolescents with NDDs.^23^ The tool covers four domain scores: physical health (seven items), psychological health (six items), social relationships (three items) and environmental health (eight items), as well as two separately scored items about an individual’s perception of their QOL (Q1) and health (Q2).^23^ Each of these items was scored from 1 to 5 on a response scale based upon a five-point Likert scale. The mean score of items within each domain was used to calculate the domain score and then the mean scores were multiplied by 4 to be directly comparable with WHOQOL-100.^23^ The domain scores exhibit a positive scaling, meaning that elevated scores are indicative of improved QOL. It was discovered that the WHOQOL-BREF scale had a high internal consistency reliability coefficient (social=0.880, psychological=0.832, environmental=0.79 and physical=0.853).^23^

The third section of the questionnaire assesses depression using the nine-item Patient Health Questionnaire (PHQ-9). PHQ-9 scores range from 0 to 27. Each of the nine items is scored from 0 (not at all) to 3 (nearly every day). A PHQ-9 score of 0–4 indicates minimal/no depression, 5–9 indicates mild depression, 10–14 indicates moderate depression, 15–19 indicates moderately severe depression and a score of 20 to 27 indicates severe depression. Furthermore, the PHQ-9 has been validated with 67% specificity and 86% sensitivity in the Ethiopian healthcare context. To screen for depression, a cut-off point of ≥10 has been employed.^24^

The fourth section of the questionnaire assesses anxiety using the Generalized Anxiety Disorder 7 (GAD-7) tool. Scores of 0, 1, 2 and 3 were assigned to the response categories of ‘not at all’, ‘several days’, ‘more than half the days’ and ‘nearly every day’, respectively, and the scores given for the seven questions were added together. Using the threshold score of 10, the GAD-7 has a sensitivity of 89% and a specificity of 82% for GAD.^25^

The fifth section of the questionnaire assesses social support using the Oslo-3 social support (OSS-3) scale. OSS-3 scores range from 3 to 14, with a score of 3–8 indicating poor social support, 9–11 moderate social support and 12–14 strong social support.^26^

The sixth section of the questionnaire asks about substance-related factors, which include history of substance use (e.g. alcohol use, khat chewing, cigarette smoking, shisha and other substances used such as cannabis) by primary caregivers. It was assessed by the alcohol, smoking and substance involvement screening tool (ASSIST), which is a brief screening questionnaire developed and validated by the WHO with an average internal consistency of kappa ranging from 0.58 up to 0.90 (for the last 3 mo) to find out about the caregiver's use of psychoactive substances. It was used to assess the current and ever substance use history of the subject.^27^

A structured questionnaire developed from various literature sources was used to assess the information provided in the seventh section of the questionnaire, which deals with chronic medical illnesses.^28–30^ ‘Have you ever had a known chronic medical condition?’ There were two possible answers to the first question: ‘Yes’ or ‘No’. If responding ‘Yes’ to the first question, then a participant was asked about the type(s) of chronic illnesses they had to determine the categories of medical illness.

### Operational definitions

QOL: based on the 26-item WHOQOL-BREF, an individual with a score approaching 100 indicates the best possible QOL, while individuals with scores close to zero have the poorest QOL.^31^

Neurodevelopmental Disorders: are a group of disorders that include intellectual developmental disorders, communication disorders, autism spectrum disorders, attention deficit hyperactivity disorder, specific learning disorders and motor disorders.^32^

A primary caregiver is defined as a person who provides care to those who have difficulties in completing tasks of daily living and thus need supervision or assistance because of some form of illness or disorder.^33^

Children and adolescents are individuals aged 2–17 y.^34^

Depression: a primary caregiver with a PHQ-9 score of ≥10 was considered to have depression.^24^

Anxiety disorder: a primary caregiver with a GAD-7 score of ≥10 was considered to have anxiety.^25^

Current substance users: using at least one specific substance (alcohol, khat, cigarettes or another substance) for non-medical purposes within the last 3 mo according to the ASSIST.^27^

Ever substance users: using at least one specific substance (alcohol, khat, cigarettes or another substance) for non-medical purposes at least once in a lifetime according to ASSIST.^27^

### Data quality control

The 1-d training was given by the principal investigator for data collectors and supervisors. Pretesting of 18 (5%) primary caregivers of children and adolescents with NDDs at Amanuel Specialized Mental Hospital was carried out to look for potential problems with the data collection tools, to confirm the consistency of the questionnaire and make any necessary adjustments. The response rate during the pretest was 100%, and some modifications, such as corrections of typing errors and rearrangements of the questionnaire (some items were reverse-coded), were made. During the pretest, the internal consistency of the tool was assessed and Cronbach's alpha was computed (physical=0.81, psychological=0.83, environmental= 0.80 and social=0.86), which was acceptable for this population. To make sure that all the required data were gathered, the principal investigator and supervisors regularly monitored and assisted the data collectors. Throughout the data collection period, principal investigators and supervisors reviewed the gathered data every day to ensure these were consistent and complete.

### Data processing and analysis

To identify outliers, missing values and inconsistencies, data were coded, recoded, cleaned and explored. The coded data were checked for accuracy before being entered into EpiData 4.6.0.2 and analyzed with SPSS version 26. The Statistical Package for Social Sciences (SPSS) version 26 from International Business Machines Corporation (IBM), incorporated in Chicago, United States of America, was used to analyze the data. In the descriptive analysis, the mean with SD, frequency and percentages were used to check the distribution of the data. Simple and multiple linear regressions were employed to identify the factors associated with the outcome variables.

Scatterplots were used to verify the linearity assumptions, the homogeneity of variances and the absence of heteroscedasticity or discernible patterns. The normality assumption was checked by the Shapiro –Wilk test and it is >0.05. The skewness ranged from –1 to ±1 and was taken as normally distributed. The Durbin–Watson statistic of 1.5–2.5 was taken as independent observations. The multicollinearity was checked and the maximum variance inflation factor reported was 1.91, which was within the acceptable level. Interaction terms were created, and they were not significant.

To find associated variables with each domain and overall QOL, simple and multivariable linear regressions were fitted for all four domains and overall QOL. For the goodness of model fit, all linear regression assumptions, adjusted R-squared (adjusted R^2^ for physical health domain=0.41, for psychological health=0.43, for social health domain=0.425 and environmental health domain=0.46), overall F-test, residual plots, SEs and outliers were considered. According to these parameters, the fitted models were good fits to explain the outcome variables. Variables with p<0.25 during the simple linear regression were selected for multivariable linear regression. p<0.05 was considered an independently associated factor for multivariable linear regressions. In multiple regression analysis, beta coefficients with 95% CIs are used to assess the degree of association and statistical significance.

## Results

### Sociodemographic and clinical characteristics of participants

Of 352 selected participants, 348 were included in the study, with a response rate of 98.8%; 241 were female (69.3%). The mean age of participants was 39.06 (SD 8.76) y, and 283 (81.3%) resided in an urban area. Also, 78.2% were married, and 191(54.9%) were orthodox Christians. Almost one-half of the study participants (47.4%) had a college education or higher, and 69 (19.8%) worked in government institutions. Furthermore, 62 (17.8%) had a medical illness; 77 (22.1%) and 53 (15.2%) had depression and anxiety symptoms, respectively (Table 1).

**Table 1.** Sociodemographic and health-related characteristics of participants among primary caregivers of children and adolescents with NDDs at Y12HMC and SPHMMC, 2022 (n=348)

Variable	Category	Number (n=348)	%
Age (y), mean±SD			39.06±7.76
Sex	Male	107	30.7
	Female	241	69.3
Religion	Orthodox	191	54.9
	Muslim	79	22.7
	Protestant	63	18.1
	Other*	15	4.3
Ethnicity	Amhara	145	41.7
	Oromo	90	25.9
	Tigrie	42	12.1
	Gurage	58	16.7
	Other**	13	3.7
Marital status	Never married	23	6.6
	Married	272	78.2
	Divorced/widowed	53	15.2
Educational status	No formal education	35	10.1
	Primary school	85	24.4
	Secondary school	63	18.1
	College and above	165	47.4
Occupational status	Governmental employee	69	19.8
	NGO employee	94	27.0
	Merchant	88	25.3
	Housewife	80	23.0
	Other***	17	4.9
Residence	Urban	283	81.3
	Rural	65	18.7
Income (in ETB)	<1539	102	29.3
	≥1539	246	70.7
Depression	Yes	77	22.1
	No	271	77.9
Anxiety	Yes	53	15.2
	No	295	84.8
Medical illness	Yes	62	17.8
	No	286	82.2
Type of medical illness (n=62)	Hypertension	26	41.9
	Diabetes mellitus	18	29.1
	Asthma	11	17.7
	Other****	7	11.3

ETB: Ethiopian birr; NDD, neurodevelopmental disorder; SPHMMC, St. Paul's Hospital Millennium Medical College; Y12HMC, Yekatit 12 Hospital Medical College.

*Catholic, Wakefata, Hawariat.

**Silte, Wolayeta, Hadia.

***student, retired, farmer.

****heart problem, cholesterol.

### Sociodemographic and clinical characteristics of children/adolescents

The children and adolescents had a mean age of 7.58 (SD 4.36) y; 263 were male (75.6%). Of the total number, 97 (27.9%), 82 (23.5%), 69 (19.8%), 58 (16.7%) and 42 (12.1%) were diagnosed as having autism spectrum disorders, attention deficit hyperactivity disorders, intellectual developmental disorders, specific learning disorders and other NDDs, respectively (Table 2).

**Table 2.** Sociodemographic and clinically related characteristics of children/adolescent participants

Variable	Category	Number (n=348)	%
Age (y), mean±SD			7.58 (4.36)
Sex	Male	263	75.6
	Female	85	24.4
Birth order	1st	102	29.3
	2nd	95	27.2
	3rd+	151	43.5
Type of diagnosis	ASD	97	27.9
	ADHD	82	23.5
	IDD	69	19.8
	SLD	58	16.7
	Other*	42	12.1
Duration of illness (y)	1–2	89	25.6
	3–4	136	39.1
	5^+^	123	35.3
Age at intervention (y)	<3	74	21.3
	3–5	125	35.9
	>5	149	42.8

ADHD: attention deficit hyperactivity disorder; ASD: autism spectrum disorder; IDD: intellectual developmental disorder; SLD: specific learning disorder.

*global developmental delay, communication disorders.

### Social support and substance-related factors of participants

Regarding social support, nearly one-half (49.7%) of participants had poor social support, 31.0% moderate social support and 19.3% strong social support. Concerning substance use, 112 participants (32.2%) consumed alcohol; 79 (22.7%) used khat; and 27 (7.8%) currently used cigarettes (Table 3).

**Table 3.** Substance use-related characteristics of study participants among primary caregivers of children and adolescents with NDDs at Y12HMC and SPHMMC, 2022 (n=348)

Variable	Category	Number (n=348)	%
Ever alcohol use	Yes	137	39.4
	No	211	60.6
Ever cigarette use	Yes	30	8.6
	No	318	91.4
Ever khat use	Yes	84	24.1
	No	264	75.9
Ever use of other substance*	Yes	13	3.7
	No	335	96.3
Current alcohol use	Yes	112	32.2
	No	236	67.8
Current cigarette use	Yes	27	7.8
	No	321	92.2
Current khat use	Yes	79	22.7
	No	269	77.3
Current use of other substance*	Yes	12	3.4
	No	336	96.6

NDD, neurodevelopmental disorder; SPHMMC, St. Paul's Hospital Millennium Medical College; Y12HMC, Yekatit 12 Hospital Medical College.

*cannabis, hashish, weed.

### Self-rated perceived QOL and health satisfaction of participants

The 26-item WHOQOL-BREF provides a QOL profile consisting of four domain scores in addition to overall perception of QOL and general health. These two last items mean that perceived QOL and general health are examined separately: question 1 asks about an individual's overall perception of QOL and question 2 asks about an individual's overall perception of general health. Each of these items was given a response scale score ranging from 1 to 5, a commonly used five-point Likert scale. Around one-third of participants (31.9%) reported that their perceived QOL was neither poor nor good, followed by 95 (27.3%) who reported that it was good. Concerning perceived satisfaction with their health, 109 (31.3%) were satisfied with their health, but 12 (3.4%) were very dissatisfied with their health (Table 4).

**Table 4.** Self-rated perceived quality of life and perceived health satisfaction among primary caregivers of children and adolescents with NDDs at Y12HMC and SPHMMC, 2022 (n=348)

	Number (n=348)	%
**Perceived quality of life**		
Very poor	19	5.5
Poor	57	16.4
Neither poor nor good	111	31.9
Good	95	27.3
Very good	66	19.0
**Perceived health satisfaction**		
Very dissatisfied	12	3.4
Dissatisfied	33	9.5
Neither satisfied nor dissatisfied	130	37.4
Satisfied	109	31.3
Very satisfied	64	18.4

NDD, neurodevelopmental disorder; SPHMMC, St. Paul's Hospital Millennium Medical College; Y12HMC, Yekatit 12 Hospital Medical College.

## QOL of respondents

In the current study, the overall mean score of QOL among primary caregivers was 62.61 (95% CI 60.56 to 63.01) ±5.17 SD. The mean scores for each domain of WHOQOL-BREF were 57.36 (95% CI 56.31 to 58.42) ±9.98 SD for the physical domain; 66.98 (95% CI 65.99 to 67.97) ±9.39 SD for the psychological domain; 66.06 (95% CI 64.70 to 67.42) ±12.91 SD for the social domain; and 60.02 (95% CI 59.06 to 60.99) ±9.14 SD for the environmental domain of QOL. In the physical health domain, 57.5% (*n*=200) and 42.5% (*n*=148) of the study participants scored below and above the mean, respectively. The lowest possible QOL is indicated by participants scoring below the mean on four WHOQOL-BREF domains. Thus, computing the percentage of participants scoring below the mean allows us to determine which of the four QOL domains is most affected. The mean is typically used as a benchmark when discussing QOL among the four WHOQOL-BREF domains. The minimum and maximum mean scores of the study participants for overall QOL were 38 and 94, respectively. Among the four domains of QOL, respondents scored the highest mean in the psychological health domain (66.98±9.39) and the lowest mean in the physical health domain (57.36±9.98) (Table 5).

**Table 5.** QOL of life among primary caregivers of children and adolescents with NDDs at Y12HMC and SPHMMC, 2022 (n=348)

Overall QOL and its domains	Mean±SD	% of participants who scored below the mean	Minimum	Maximum	95% CI
Physical	57.36 ± 9.98	57.5%	31.00	81.00	(56.31, 58.42)
Psychological	66.98 ± 9.39	37.4%	38.00	94.00	(65.99, 67.97)
Social	66.06 ± 12.91	36.5%	19.00	94.00	(64.70, 67.42)
Environmental	60.02 ± 9.14	48.6%	31.00	81.00	(59.06, 60.99)
Overall QOL	62.61 ± 5.17	35.3%	37.50	76.75	(60.56, 63.01)

NDD, neurodevelopmental disorder; QOL, quality of life; SPHMMC, St. Paul's Hospital Millennium Medical College; Y12HMC, Yekatit 12 Hospital Medical College.

## Factors associated with QOL

Simple linear regression analysis was carried out between the WHO-BREF domains and each independent variable. Variables with p<0.25 during simple linear regression analysis were selected for further analysis in multiple linear regression analysis. Age, no formal education, average monthly income, current alcohol use, depression symptoms and the presence of medical illness were factors significantly associated with overall QOL and its domains. For the psychological health domain, being divorced/widowed, current khat use, being a housewife, average monthly income, the presence of anxiety disorders and poor social support were negatively associated factors. For the social health domain, being divorced or widowed, occupational status, the presence of depression and poor social support were significantly associated factors. The environmental health domain was found to be significantly associated with the factors of age, monthly income, lack of formal education and the presence of medical illness.

A unit increase in age decreased the physical health domain of QOL of primary caregivers by 0.25-fold (β=−0.25, 95% CI −0.43 to −0.07, p=0.001). Respondents who had no formal education had about 3.34-fold decreased physical health domain QOL (β=−3.34, 95% CI −6.23 to 2.17, p=0.011) compared with those who had attended college and above. In comparison with primary caregivers with no depressive symptoms, those with depressive symptoms experienced a 6.32-fold decrease in the physical health domain (β=−6.32, 95% CI −11.96 to −0.67, p=0.02). Primary caregivers who have a medical illness experienced a 4.32-fold decrease in the physical health domain (β=−4.32, 95% CI −7.64 to −2.91, p=0.001) compared with those without a medical illness.

An individual who was divorced/widowed had about a 2.99-fold decreased psychological health domain QOL compared with those who were married (β=−2.99, 95% CI −5.82 to −0.17, p=0.03). Compared with a government employee, being a housewife decreased the psychological health domain QOL by 4.32-fold (β=−4.32, 95% CI −7.56 to −1.07, p=0.01). Compared with primary caregivers with no anxiety symptoms, primary caregivers with anxiety symptoms experienced a 3.07-fold decrease in the psychological health domain QOL (β=−3.07, 95% CI −5.80 to −0.34, p=0.03), while individuals with poor social support had a psychological health domain QOL that decreased 3.25 times (β=−3.25, 95% CI −5.89 to −0.61, p=0.01) compared with those who had strong social support.

Being divorced or widowed resulted in about 0.62-fold less social health domain QOL (β=−0.62, 95% CI −1.33 to −0.10, p=0.02) compared with those who were married, and individuals who had poor social support experienced 3.39-fold decreased social health domain QOL (β=−3.39, 95% CI −11.30 to −4.61, p=0.02) compared with those who had strong social support.

Primary caregivers with a medical illness had an environmental health domain QOL that decreased 3.11-fold (β=−3.11, 95% CI −5.71 to −0.51, p=0.01) compared with those without medical illness, while primary caregivers with an average monthly income of <1539 Ethiopian birr (ETB) had about a 2.25-fold decreased environmental health domain QOL (β=−2.25, 95% CI −4.37 to −0.12, p=0.03) compared with those with an average monthly income of ≥1539 ETB. Compared with being a government employee, being an employee of a non-governmental organization resulted in a decrease in environmental health domain QOL of 2.60-fold (β=−2.60, 95% CI −5.17 to −0.03, p=0.04), while a unit increase in age in a primary caregiver resulted in a decrease in their environmental health domain QOL of 4.57-fold (β=−4.57, 95% CI −9.06 to −0.09, p=0.04) (Table 6).

**Table 6.** Multiple linear regression models for physical, psychological, social and environmental domains of quality of life among primary caregivers of children and adolescents in Addis Ababa, Ethiopia, 2022 (n=348)

	Adjusted β (95% CI)			
Variable	Physical health	Psychological health	Social health	Environmental health
Age	−0.25 (−0.43, −0.07)*	−2.05 (−6.85, 2 .75)	−4.83 (11.18, 1.50)	−4.5 (−9.06, 0.09)*
Marital status Married Never married Divorced/widowed	1.00 −1.11 (−5.48, 3.26) −1.53 (−4.64, 1.57)	1.00 2.04 (−1.96, 6.05) −2.99 (−5.82, −0.17)*	1.00 4.20 (−1.43, 9.83) −0.6 (−1.3, −0.10)*	1.00 2.32 (−1.64, 6.29) −1.60 (−4.42, 1.2)
Educational status No formal education Primary school Secondary school College and above	−3.34 (−6.23, −2.17)* −0.58 (−3.23, 2.06) −1.82 (−4.71, 1.05) 1.00	−0.17 (−3.51, 3.17) −1.31 (−3.74, 1.13) −1.10 (−3.75, 1.54) 1.00	0.92 (−3.76, 5.61) −1.47 (−4.87, 1.93) −1.17 (−4.88, 2.53) 1.00	1.67 (−1.62, 4.98) −0.93 (−3.3, 1.46) −0.61 (−3.2, 2.00) 1.00
Occupational status Governmental employee NGO employee Merchant Housewife Other	1.00 −0.96 (−3.78, 1.85) 0.48 (−2.80, 3.77) 0.96 (−4.48, 2.55) 3.17 (−2.25, 8.60)	1.00 1.98 (−0.60, 4.56) 4.18 (1.15, 7.20) −4.32 (−7.56, −1.07)** −6.84 (−11.84, −1.85)*	1.00 3.97 (0.35, 7.59)* −1.42 (−5.66, 2.81) 3.67 (−0.85, 8.20) −3.83 (10.82, 3.16)	1.00 −2.6 (−5.2, −0.03)* −2.75 (−5.7, 0.23) −0.90 (−4.1, 2.29) −3.92 (−8.8, 0.99)
Monthly income (in ETB) <1539 ≥1539	1.00 0.71 (−0.18, 4.43)	1.00 2.95 (0.79, 5.10)*	1.00 0.24 (−4.79, 1.21)	−2.3 (−4.4, −0.12)* 1.00
Depression Yes No	−6.3 (−11.9, −0.67)** 1.00	−1.76 (−4.14, 0.62) 1.00	1.85 (−1.47, 5.17) 1.00	−0.83 (−3.2, 1.51) 1.00
Anxiety Yes No	2.24 (−0.72, 5.21) 1.00	−3.07 (−5.80, −0.34)*** 1.00	−0.67 (−4.49, 3.15) 1.00	0.08 (−2.61, 2.77) 1.00
Medical illness Yes No	−4.32 (−7.64, −2.91)** 1.00	0.81 (−1.82, 3.43) 1.00	1.75 (−1.93, 5.45) 1.00	−3.11 (−5.7, −0.5)* 1.00
Social support Poor Moderate Strong	−2.40 (−5.28, 0.47) 8.62 (3.15, 14.09)** 1.00	−3.25 (−5.89, −0.61)** 2.31 (−0.52, 5.16) 1.00	−3.4 (−11.3, 4.6)* −2.38 (−6.37, 1.59) 1.00	0.23 (−2.35, 2.81) −1.08 (−3.8, 1.71) 1.00

ETB: Ethiopian birr; NGO, non-governmental organization.

1.00=reference.

*p<0.05.

**p<0.01.

***p<0.001.

Adjusted R-squared=0.41, 0.43, 0.425 and 0.46 for physical, psychological, social and environmental, respectively.

## Discussion

This study aimed to assess the overall QOL profile with its domains and associated factors among primary caregivers of children and adolescents with NDDs attending outpatient department services at Y12HMC or SPHMMC, Addis Ababa Ethiopia. The majority of parents of children and adolescents with neurodevelopmental abnormalities in Ethiopia, as in other impoverished remote locations, do not receive attention from trained attendants. Additionally, these primary caregivers deal with associated difficulties such as physical, emotional, social and environmental problems that impair their QOL.

In this study, the mean overall QOL score among study participants was 62.60 (SD 8.35). This study result was in line with studies conducted in China, Jordan and Ireland.^35–37^ In the current study, the mean overall QOL of primary caregivers was higher compared with studies held in Belgium, Taiwan, Nigeria and South Africa.^11,38–40^ This discrepancy can potentially be attributed to sample size differences, the clinical characteristics of the participants, sociocultural differences and the study setting.^40–43^ The current study sample size was higher compared with those in Taiwan, Nigeria and Belgium. In the Taiwan study, participants’ substance use (especially alcohol) was higher (43.9%) compared with the current study (32.2%). Different studies show that primary caregivers using substances had a lower QOL^41–43^ than non-users. More than one-half (60%) of study participants in Belgium had a psychiatric comorbidity and about one-third (31.5%) had a medical comorbidity compared with 37.3% and 17.8% in the current study, respectively. These high numbers of psychiatric disorders and medical illness might contribute to low overall mean QOL.

Nevertheless, this finding was lower than the mean overall QOL score of other countries, such as Bahrain, Pakistan, USA, China and the UK.^36,44–47^ Possible reasons for this variation in QOL include differences in the clinical characteristics of the study participants, sample size, social support and lifestyles of the caregivers compared with the current study. The sample sizes in the studies performed in USA, Taiwan and the UK were higher compared with the one in this study. Compared with the current study's rate of medical illness (17.8%), study participants from Pakistan had lower medical illness rates (5.95%). While the strong social support factor of study participants was 19.3% in the current study, research conducted in the UK and USA showed higher levels of strong social support (32% and 42.4%, respectively).

Concerning the domains of QOL, the physical health domain had a low mean score in QOL (57.36), which indicated that the physical health domain of study participants was the most affected. Previous studies conducted in USA and India reported similar results, showing that the physical health domain of QOL of primary caregivers was the most affected.^20,48^ This consistency of low scores in the physical health domain of QOL of primary caregivers of children and adolescents with NDDs could be caused by caring for children with NDDs, restricting them from performing daily living activities, as well as losing energy and sleep related to caring for those children and adolescents. On the contrary, results from studies in Australia, the UK and Brazil showed better physical health and the lowest mean score in the social domain.^28,46,49^ The differences among mean QOL domain scores could be a result of variations in beliefs, cultures and lifestyle factors that affect QOL measures. Because QOL is a subjective concept, it may be perceived differently in people of different cultures, beliefs and values.

In the current study, age, educational status, marital status, occupational status and average monthly income were among the sociodemographic factors that had a significant association with the overall QOL and its domains in primary caregivers of children and adolescents with NDDs. Age was negatively associated with the physical health domain of QOL (β =−0.25, 95% CI −0.43 to –0.07). This is in line with other research, which indicated that age was negatively associated with physical health domain QOL.^35^ Such findings may reflect that younger people are more likely to enjoy better health than the elderly and, in addition, aging may diminish the physiological system, which could limit the different activities of the body.

This study indicated that no formal education was negatively associated with a lower score of physical health domain QOL (β=−3.34, 95% CI −6.23 to −2.17), indicating that primary caregivers of children and adolescents with NDDs who had no formal education have a lower understanding of disorders, treatment and outcomes, as well as being unable to make decisions on better self-care. This is inconsistent with the study finding from Saud Arabia, where educational status had no significant association with QOL.^50^ This difference could be because of the differences in sociodemographic status among participants.

Being a divorced/widowed primary caregiver of a child or adolescent with a NDD was negatively associated with psychological health domain (β=−2.99, 95% CI −5.82 to −0.17) and social domain QOL (β=−0.62, 95% CI −1.3 to −0.10). Study findings from other parts of the world are in line with results from the current study.^15,19,50,51^ One possible explanation for this could be that divorced or widowed primary caregivers are psychologically unstable and more likely to experience negative feelings, lower self-esteem, behavioral problems, anxiety, depression and other mood disorders that in turn lead to poor QOL compared with those who are married.

The current study indicates that average monthly income was negatively associated with the environmental health domain (β= −2.25, 95% CI −4.4 to −0.12). However, other studies showed no associations between QOL and primary caregivers’ monthly income.^11,40^ In the other study, income was only reported on the mean annual income of caregivers. However, primary caregivers’ methods of disclosing their income levels might not be accurate, especially for unsalaried primary caregivers. This might overestimate the association.

Poor social support was associated with a lower score in the psychological domain (β= −3.25, 95% CI −5.89 to −0.61) and social domain (β=−3.39, 95% CI −11.3 to −4.6), while moderate social support was associated with better scores in the physical health domain (β=8.62, 95% CI 3.15 to 14.09) compared with poor social support. This study finding was supported by a study carried out in Bangladesh.^15^ One possible explanation for this could be the feeling of being loved and wanting to contribute towards providing a supportive environment that helps to cope with the challenges of providing care for children and adolescents with NDDs.

Furthermore, in this study, having a chronic medical illness was negatively associated with the physical health domain (β=−4.32, 95% CI −7.64 to −2.91) and environmental health domain (β=−3.11, 95% CI −5.7 to −0.5). This result was consistent with previous studies performed in Brazil, South Africa and Malaysia.^11,28,52^

The results of this study also revealed that having a psychiatric disorder like anxiety (β=−3.07, 95% CI −5.80 to −0.34) or depression (β=−6.32, 95% CI−11.96 to –0.67) was negatively associated with the psychological and physical health domains, respectively. This result is supported by an earlier study conducted in Australia, which indicated that psychiatric disorders were negatively associated with the overall QOL and its domain.^6^ This could be explained by having to depend on different medications, the money needed to afford those drugs, and the demand for healthcare services because they have comorbid conditions, contributing to impairment of their psychological health and overall QOL. Another possible explanation could be depressed mood, hopelessness and loss of interest exerting a profound and negative effect on QOL. It could also be caused by the impact of depression and anxiety symptoms on the primary caregiver's perception of their child’s disorders.

## Strengths and limitations

The main strength of the current study is that it used standardized cross-culturally validated tools to measure QOL and other variables. In addition, appropriate translation of the questionnaire and proper supervision of the data collection were carried out. However, because it had a cross-sectional study design, it is not possible to establish a temporal relationship between QOL and its associated factors.

## Conclusions and recommendations

The findings from this study indicate that all dimensions of QOL of primary caregivers of children and adolescents with NDDs in this study setting were compromised. According to the results of the current study, the physical health domain was most negatively affected because caring for children and adolescents with NDDs causes disruption to caregivers’ daily activities, depletes their energy, causes a build-up of fatigue, limits their mobility and alters their sleep patterns. Being divorced or widowed, a lack of formal education, age, average monthly income, poor social support, depression, anxiety and the presence of medical illness are factors associated with QOL in all domains.

Healthcare providers need to provide psychosocial intervention and screening for primary caregivers of children and adolescents with NDDs. Healthcare providers require training to be able to identify and manage the symptoms of depression, anxiety and medical illness. It would have been better to conduct a prospective study to investigate the cause-and-effect relationship between risk factors of QOL among primary caregivers of children and adolescents with NDDs.

## Data Availability

All the data included in the manuscript can be accessed from the corresponding author Jerman Dereje upon request by email (jermandereje82@gmail.com).
